# Diagnostic and prognostic values of HCG15 and morrbid in acute myocardial infarction

**DOI:** 10.3389/fphar.2024.1492746

**Published:** 2024-11-25

**Authors:** Min Huang, Bohua Wu, Xiuxia Ou, Shuo Sun, Kedong Han, Lijian Li, Haiyan Liang, Chunchan Qiu, Qingbo Xu

**Affiliations:** ^1^ Department of Cardiology, Maoming People’s Hospital, Maoming, Guangdong, China; ^2^ Department of Cardiology, Huazhou People’s Hospital, Maoming, Guangdong, China; ^3^ Department of Cardiology, Guangdong Provincial People’s Hospital, Guangzhou, Guangdong, China

**Keywords:** myocardial infarction, HCG15, morrbid, diagnosis, prognosis

## Abstract

**Background:**

Acute myocardial infarction (AMI) represents the gravest manifestation of ischemic heart disease, with the primary cause of mortality and morbidity worldwide. Although timely and accurate diagnosis of AMI is crucial in clinical practice, they are impeded by the limitation of current biomarkers. We aimed to explore the potential predictive value of two novel long non-coding RNA (lncRNA) HCG15 and Morrbid in AMI diagnosis and prognosis.

**Method:**

We measured the lncRNA levels in the blood samples of 412 AMI patients and 111 healthy volunteers with the RT-PCR method. Receiver operating characteristic (ROC) curves were plotted to access the diagnostic value of selected lncRNAs. Restricted cubic splines (RCS) and the Kaplan-Meier method were utilized to examine the predictive value of the selected lncRNAs in AMI diagnosis.

**Result:**

ROC curves identified an acceptable diagnostic value of HCG15 and Morrbid (AUC for HCG15: 0.937; AUC for Morrbid: 0.940). RCS and Kaplan-Meier analysis revealed the cut-off value of 3.6 for HCG15 and 4.0 for Morrbid have a good predictive value in MACCE within 12 months once AMI was diagnosed (*p*-value for HCG15: *p* = 0.025; *p*-value for Morrbid: *p* < 0.0001).

**Conclusion:**

HCG15 and Morrbid were confirmed as promising lncRNA biomarkers for both diagnosis and prognosis of AMI in this study. Additionally, their importance of application in real-world clinical practice and underlying mechanisms in AMI diagnosis and prognosis remain to be explored.

## Introduction

According to the Global Burden of Disease (GBD) study, ischemic heart disease (IHD) remained the leading cause of mortality and morbidity globally, accounting for 9,440,000 deaths and 185,000,000 disability-adjusted life years in 2021 (Vaduganathan et al., 2022). Acute myocardial infarction (AMI) is often deemed the most severe manifestation of IHD due to its rapid and irreversible damage to cardiomyocytes (Mechanic OJ and Grossman, 2024). Within the past decades, progressive treatment of cardiovascular risk factors and improvement in healthcare system facilitated a slow but gradual reduction of mortality rate (Nowbar et al., 2019). However, along with this slow decline of AMI mortality, non-fatal events occurred post-survival of AMI (i.e., heart failure, stroke, and bleeding) have incidentally increased (Shah et al., 2021), further deteriorating patients’ quality of life and intensifying the indirect economic burden on society (Weintraub et al., 2011). Therefore, early and accurate diagnosis of AMI is crucial for subsequent revascularization therapy, bringing substantial benefits for both individuals and the society.

Long noncoding RNAs (lncRNAs) have been demonstrated to participate multifarious cardiomyocyte activities, including genomic imprinting, epigenetic regulation, cell proliferation, development, aging, and apoptosis (Wang and Sun, 2020). During the occurrence of AMI, functional impairment of cardiomyocytes may cause the release of heart-specific lncRNAs. In this regard, lncRNAs exhibited promising potential in assisting the diagnosis and therapeutic targets of AMI. HLA complex group 15 (HCG15), one of the differentially expressed lncRNA, was proved to be encapsulated in exosomes released by cardiomyocytes during hypoxia and involved in cardiomyocyte apoptosis, inflammatory response, and proliferation inhibition through the activation of the NF-κB/p65 and p38 pathways (Lin et al., 2021). Another lncRNA named myeloid RNA regulator of Bim-induced death (Morrbid) was initially identified as a leukocyte-specific lncRNA and plays a key role in controlling leukocyte lifespan (Kotzin et al., 2016). Recently, Morrbid was also identified to be overexpressed in the cardiomyocytes with hypoxia or oxidative stress, potentially protecting the heart from AMI via antiapoptosis through its target gene serpine1 (Yu et al., 2023). However, the analysis of the correlation between those lncRNAs and AMI remained in the laboratory settings. Clinical evidence supporting the diagnostic and prognostic value of HCG15 and Morrbid in AMI is currently lacking.

This study aimed at evaluating the potential diagnostic and prognostic value of HCG15 and Morrbid in AMI management to introduce potential novel biomarkers for application in clinical practice.

## Materials and methods

### Study population

From January 2021 to July 2022, a total of 412 consecutive patients with AMI were recruited from Maoming People’s Hospital in Guangdong, China. Additionally, 111 volunteers without a history of cardiovascular diseases or other essential organ diseases were randomly recruited as healthy controls. The inclusion criteria were as followed: i) clinically diagnosed with AMI for AMI group aligned with the AHA and ESC guideline (Byrne et al., 2023; Lawton et al., 2021), and ii) without a history of any cardiovascular disease for control group. AMI patients who met the following criteria were excluded in the study: i) those with concurrent advanced or malignant diseases (such as organ failure or cancer), and ii) those who declined to participate. Data regarding age, body mass index (BMI), gender, hypertension, statin treatment, and diabetes were collected through history-taking and physical examination. The levels of specific lncRNAs were measured using venous blood samples in the hospital laboratory after enrollment. This study was approved by the ethical committee of Maoming People’s Hospital (Approval No. PJ2022MI- LXQ030-01), and all participants signed informed consent forms to participate in this study.

### Blood sample collection

Blood samples were obtained from AMI patients immediately after presentation and within 6 h of symptom onset. Following routine laboratory tests for AMI diagnosis, the remaining blood samples from the EDTA tube, free from anticoagulant and hemolysis, were collected. The peripheral blood samples were centrifuged at 3,500 rpm for 10 min, and the resulting supernatant was transferred to an RNase-free tube and stored at −80°C for subsequent analyses. Plasma levels were measured for all potential biomarkers.

### RNA extraction and qRT-PCR analysis

RNA was extracted from plasma using the miRNeasy Mini Kit (Qiagen, #217004). iScript^®^ cDNA Synthesis Kit (Bio-Rad, #170–8,891) was utilized for the reverse transcription of cDNA. RNase-Free DNase I Kit (Norgen, #25710) was utilized for on-column DNA removal to mitigate the potential genomic DNA contamination. The One Step TB Green^®^ PrimeScript™ RT-PCR Kit II (Perfect Real Time) (TaKaRa, #RR086A) was utilized in the qRT-PCR procedure, employing specifically designed primers targeting lncRNA HCG15 and Morrbid. The aforementioned experiments were conducted in compliance with the instructions provided for the corresponding reagents. The internal control for qRT-PCR analysis of HCG15 and Morrbid was designated as GAPDH. The specific primers used in this studies were as follows: HCG15, 5′- CGC​GGG​TCA​CCT​TCT​GAA​TTT-3′ (forward), 5′- AAA​GAG​CGC​AGT​CCT​TGC​TG-3 ′ (reverse) and Morrbid, 5′- ACT​GGA​TGG​TCG​CTG​CTT​TT-3’ (forward), 5′-CTT​CCC​AGG​AAC​TGT​GCT​GT-3’ (reverse). All tests were performed in triplicate with calculation of mean for the following analyses. The relative expression level of selected lncRNA was calculated following the 2^−△△Ct^ method.

### Clinical outcomes

The primary endpoint to test the prognostic value was major adverse cardiac and cerebrovascular events (MACCE), including all-cause mortality, non-procedural myocardial infarction, repeat revascularization, and stroke (Mäkikallio et al., 2016).

### Statistical analysis

Continuous variables were presented with median (interquartile range, IQR) and compared with the Kruskal–Wallis test. Categorical variables were presented with counts (percentage) and compared with the χ2 test. Receiver operating characteristic (ROC) curves were plotted and evaluated with the area under the ROC curve (AUC) to assess the diagnostic power of selected lncRNAs in AMI. Restricted cubic splines (RCS) were employed to visualize the relationship between the level of selected lncRNAs and the occurrence of MACCE within 12 months after AMI diagnosis. Additionally, cut-off values of the selected lncRNAs with respect to the hazard ratio (HR) of 1.00 were determined in RCS for further analysis. Survival analyses were performed using the Kaplan-Meier method followed by the log-rank test to compare the MACCE occurrences within 12 months between different levels of selected lncRNAs. All analyses and data visualization were conducted using the R (version 4.2.1), and a two-sided *p* < 0.05 was considered to be statistically significant.

## Results

### Baseline characteristics

A total of 523 individuals were eventually included in this study, with 412 AMI patients and 111 healthy controls. The median age of AMI group and control group was 66.28 (IQR, 62.69–69.75) years and 65.54 (IQR, 63.27–68.62) years, respectively. There was no difference between these two groups, with 53.6% of males in the AMI group and 52.3% of males in the control group. The BMI level was significantly higher in the AMI group compared to the control group [27.09 (25.06–29.13) vs 22.02 (20.25–23.88), *p* < .001]. The prevalence of hypertension, statin treatment, and diabetes was higher in the AMI group than in the control group (hypertension, *p* < 0.001; statin treatment, *p* < 0.001; and diabetes, *p* = 0.005). Other detailed information about the baseline characteristics was shown in Table 1.

**TABLE 1:** Baseline characteristics of the cohort.

Variables	AMI (N = 412)	Control (N = 111)	*p*-value
Age, years	66.28 (62.69–69.75)	65.54 (63.27–68.62)	.406
BMI, kg/m^2^	27.09 (25.06–29.13)	22.02 (20.25–23.88)	<.001
Male	221 (53.6%)	58 (52.3%)	.878
Hypertension	265 (64.3%)	42 (37.8%)	<.001
Statin treatment	214 (51.9%)	24 (21.6%)	<.001
Diabetes	62 (15%)	5 (4.5%)	.005

Data were presented as median (interquartile range) or counts (percentage). Abbreviation: AMI, acute myocardial infarction; BMI, body mass index.

### The diagnostic value of HCG15 and morrbid

**FIGURE 1:**
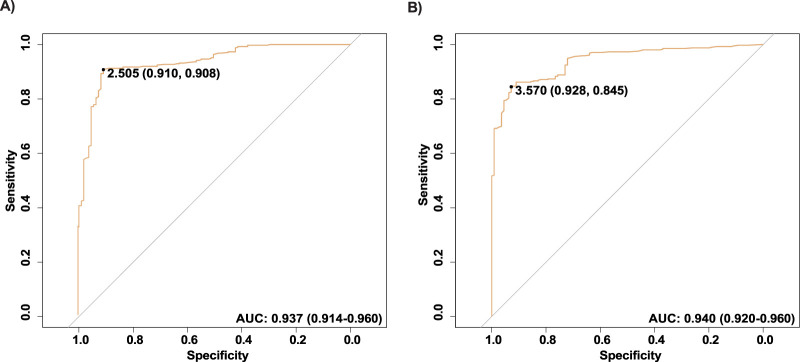
ROC of the selected lncRNAs. **(A)** The ROC curve of HCG15; **(B)** The ROC curve of Morrbid. ROC, Receiver operating characteristic.

To evaluate the diagnostic value of HCG15 and Morrbid in AMI, we presented the ROC curves in Figure 1. The AUC of HCG15 in AMI diagnosis was 0.937 (95% CI, 0.914–0.960), and the cut-off value of the relative expression level was 2.505, with a specificity of 0.910 and a sensitivity of 0.908, respectively (Figure 1A). The AUC of Morrbid in AMI diagnosis was slightly higher than HCG15 of 0.940 (95% CI, 0.929–0.960), and the cut-off value of the relative expression level was 3.570, with a specificity of 0.928 and a sensitivity of 0.845, respectively (Figure 1B).

## The non-linear association based on RCS logistic regression

**FIGURE 2:**
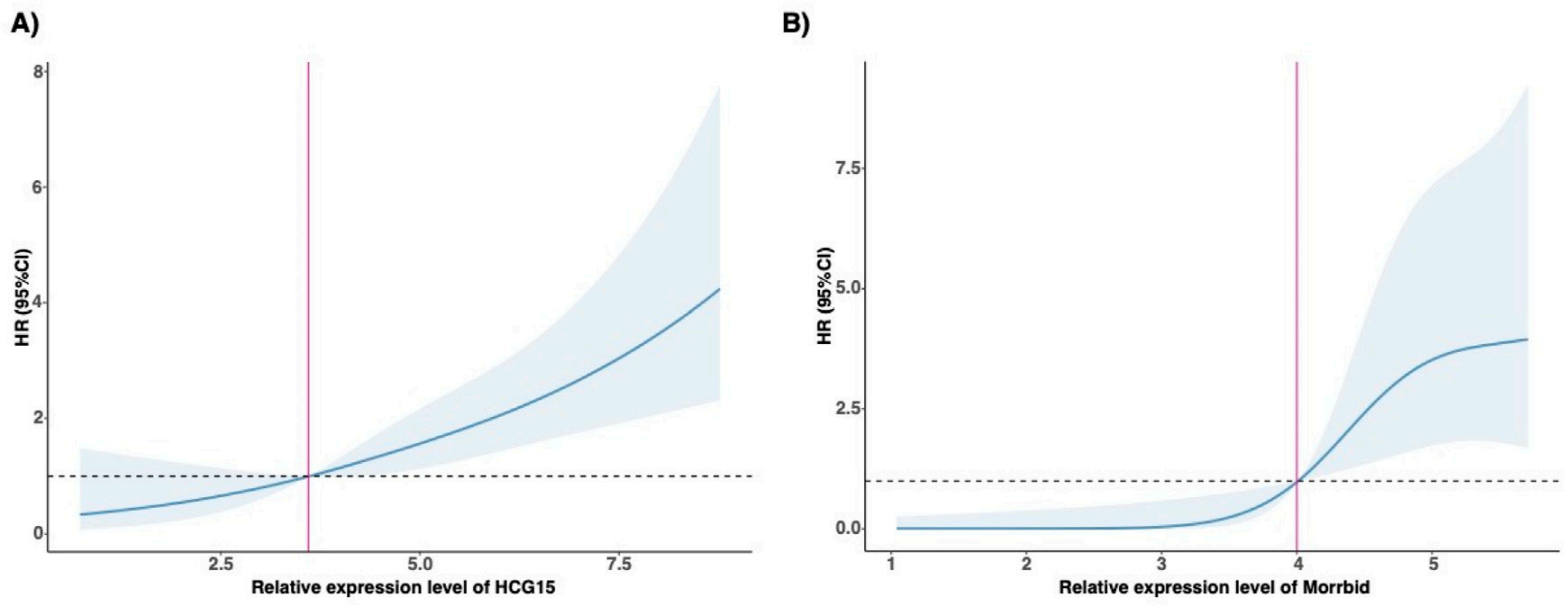
Association between relative expression level of the selected lncRNAs and HR of MACCE. **(A)** RCS of HCG15; **(B)** RCS of Morrbid. HR, harzard ratio; MACCE, major adverse cardiovascular and cerebrovascular event; RCS, restricted cubic spline.

Figure 2A exhibited a non-linear association between the hazard ratio of MACCE within 12 months and the relative expression level of HCG15 among AMI patients. The relative expression level of HCG15 associated with a hazard ratio of more than 1.00 for MACCE within 12 months was 3.6. Figure 2B similarly showed a non-linear association between the hazard ratio of MACCE within 12 months and the relative expression level of Morrbid. The threshold of the relative expression level of Morrbid associated with a hazard ratio of 1.00 of MACCE within 12 months was 4.0.

### The prognostic value of HCG15 and morrbid for MACCE

**FIGURE 3:**
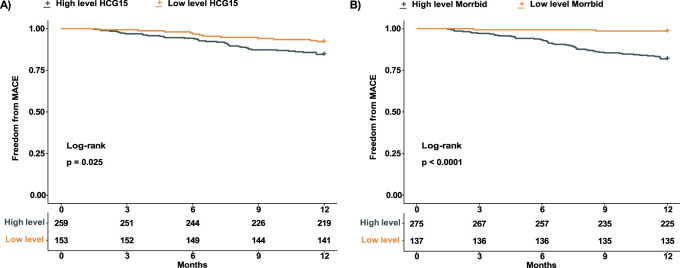
Kaplan-Meier curve of AMI patients with different expression level. **(A)** Kaplan- Meier curve of patients with different HCG15 expression level; **(B)** Kaplan-Meier curve of patients with different Morrbid expression level. AMI, acute myocardial infarction.

A total of 52 patients experienced MACCE during the 12 months follow-up. We tested the association between expression level of HCG15 and Morrbid with the occurrence of MACCE, with the visualization in Figure 2. According to the RCS regression models, patients with AMI were divided into a high-level HCG15 group and a low-level HCG15 group with a cut-off expression level of 3.6 as well as a high-level Morrbid group and a low-level Morrbid group with a cut-off expression level of 4.0. To evaluate the prognostic value of HCG15 and Morrbid in AMI, we presented the Kaplan- Meier curves for freedom from MACCE in different levels of HCG15 and Morrbid in Figure 3. The occurrence of MACCE was significantly higher in the high-level HCG15 group than in the low-level HCG15 group (*p* = 0.025) (Figure 3A). Similarly, a higher occurrence of MACCE was observed in the high-level Morrbid group (*p* < 0.001) (Figure 3B).

## Discussion

Timely and accurate diagnosis of AMI is of great importance to maximize the benefits of treatment and further prognosis. To date, the rapid rule-in and rule-out of suspected AMI cases without apparent abnormalities in the ECG presentation in clinical practice relies on the elevated level of high-sensitivity cardiac troponin (hs-cTn) within 0 h/1 h algorithm above the upper limit of 99th among health population (Byrne et al., 2023). However, the diagnostic value of hs-cTn is confounded by general background (e.g., age, renal dysfunction, time from chest pain onset, and sex) (Byrne et al., 2023), other myocardial injury conditions (e.g., myocarditis, chronic heart failure, and cardiomyopathy) (Park et al., 2017; Ammirati et al., 2020), and extra-cardiac disease (e.g., diabetes mellitus, hypertension, and stroke) (Agewall et al., 2011), thereby challenging differential diagnosis of AMI in some particular patient status. An optimal biomarker should possess high specificity and sensitivity for disease diagnosis, providing timely and cost-effective results that hold significance. Furthermore, it should offer additional value beyond existing markers or clinical characteristics, and potentially even have the ability to predict prognosis (Chan and Ng, 2010). However, ideal biomarkers are still warrant in clinical practice.

Recently, mounting evidence has uncovered the potential predictive value of lncRNAs in AMI diagnosis (Zhu et al., 2023; Xie et al., 2022), given their widespread involvement in roles of normal cardiomyocyte activities (Wang and Sun, 2020). In this study, we assessed the potential diagnostic utility of HCG15 and Morrbid in cases of AMI due to their potential higher cardiac specificity and may be less disturbed by individual profiles, initial suggested in animal and cellular models (Lin et al., 2021; Yu et al., 2023). We also examined their predictive value for MACCE within 12 months following the diagnosis of AMI. Ultimately, this study revealed the potential supplementary diagnosis and predictive value of HCG15 and Morrbid in AMI management with real-world data, which might provide novel insight in AMI.

HCG15 was suggested to be the potential novel biomarker associated with AMI (Lin et al., 2021), and this hypothesis was further verified by our results. Cardiomyocyte apoptosis and inflammatory reaction are involved in the process of AMI with the activation of multiple signaling pathways. For instance, the mitogen-activated protein kinase (MAPK) signaling pathway and its subunit p38 induce cardiomyocyte apoptosis, dysfunction, and fibrosis (Ding et al., 2018). Moreover, the activation of NF-κB and its subunit signaling pathway can regulate the expression of inflammatory cytokines, which are involved in the development of AMI (Stokes and Winn, 2014). A previous study confirmed that HCG15 was higher among AMI patients compared with health individuals and participated in apoptosis, inflammatory reaction, and proliferation inhibition of cardiomyocytes by activating the p38 and NF-κB/p65 pathways (Lin et al., 2021). In addition, for the first time to our knowledge, we indicated the prognostic value of HCG15 in predicting the MACCE within 12 months. This may be explained by the involvement of HCG in cardiomyocyte apoptosis, indirectly reflecting the extent of myocardial fibrosis, which is associated with malignant arrhythmias and chronic heart failure. However, this theory requires further verification in future studies.

Morrbid was initially identified as a leukocyte-specific lncRNA that existed in both mice and humans to regulate leukocyte lifespan with its anti-apoptotic effect (Kotzin et al., 2016). After that, its association with left ventricular hypertrophy and AMI was further discovered (Yu et al., 2023; Zhang et al., 2015). Cardiac Morrbid levels increase during the pathophysiological process of AMI, such as hypoxia stress and oxidative stress, exerting an anti-apoptotic effect on cardiomyocytes to protect against infarct enlargement and cardiac dysfunction (Yu et al., 2023). However, our findings revealed that a higher risk of MACCE within 12 months was predicted by a higher level of Morrbid just after presentation to the hospital. Indeed, leucocytes play a central role in the inflammatory response of cardiomyocytes in AMI (Naruko et al., 2002; Sasmita et al., 2022), and increased Morrbid expression in leukocytes may prolong and intensify the inflammatory response, contributing to more severe cardiac injury and adverse clinical outcomes. Similarly, this hypothesis warrants further validation in future studies.

Several limitations should be addressed in this study. First, selective bias inevitably existed in this study due to some AMI patients refusing to participate. Therefore, multicenter studies with large sample sizes are warranted to validate our pioneering results. Second, informative or recall bias also occurred in this study because of the history taking of medical and medication history. Future studies should incorporate additional objective evidence, such as the comorbidity with diabetes being defined by the elevated level of fasting blood glucose or glycated hemoglobin. Third, the potential involvement of these two lncRNAs in other pathological processes needs to be more adequately considered. Hence, it is crucial to interpret the results of this study with caution. Last, although this study revealed the association between two lncRNAs and the value of diagnosis and prognosis in AMI, the underlying mechanisms of that remain to be elucidated.

## Conclusion

In this study, we investigated the predictive value of HCG15 and Morrbid in AMI diagnosis, revealing that both of them were potential biomarkers for AMI diagnosis. In addition, we identified, for the first time, their predictive capability for MACCE within 12 months after AMI diagnosis. Conclusively, HCG15 and Morrbid emerge as novel and promising biomarkers associated with AMI, warranting further research focusing on them.

## Data Availability

The raw data supporting the conclusions of this article will be made available by the authors, without undue reservation.
